# Symbiont-mediated protection varies with wasp genotype in the *Drosophila melanogaster*–*Spiroplasma* interaction

**DOI:** 10.1038/s41437-019-0291-2

**Published:** 2020-01-02

**Authors:** Jordan Elouise Jones, Gregory David Douglas Hurst

**Affiliations:** Institute of Integrative Biology, University of Liverpool, Liverpool, L69 7ZB UK

**Keywords:** Evolutionary ecology, Evolutionary genetics

## Abstract

The ability of an insect to survive attack by natural enemies can be modulated by the presence of defensive symbionts. Study of aphid–symbiont–enemy interactions has indicated that protection may depend on the interplay of symbiont, host and attacking parasite genotypes. However, the importance of these interactions is poorly understood outside of this model system. Here, we study interactions within a *Drosophila* model system, in which *Spiroplasma* protect their host against parasitoid wasps and nematodes. We examine whether the strength of protection conferred by *Spiroplasma* to its host, *Drosophila melanogaster* varies with strain of attacking *Leptopilina heterotoma* wasp. We perform this analysis in the presence and absence of ethanol, an environmental factor that also impacts the outcome of parasitism. We observed that *Spiroplasma* killed all strains of wasp. However, the protection produced by *Spiroplasma* following wasp attack depended on wasp strain. A composite measure of protection, including both the chance of the fly surviving attack and the relative fecundity/fertility of the survivors, varied from a <4% positive effect of the symbiont following attack of the fly host by the Lh14 strain of wasp to 21% for the Lh-Fr strain in the absence of ethanol. We also observed that environmental ethanol altered the pattern of protection against wasp strains. These data indicate that the dynamics of the *Spiroplasma*–*Drosophila*–wasp tripartite interaction depend upon the genetic diversity within the attacking wasp population, and that prediction of symbiont dynamics in natural systems will thus require analysis across natural enemy genotypes and levels of environmental ethanol.

## Introduction

All organisms face a threat from natural enemies and, in response, are typically able to defend themselves through a variety of protective mechanisms. In many species, the outcome of an encounter may in part be determined by defensive symbionts within the host (and indeed offensive symbionts in the natural enemy) (Brownlie and Johnson 2009; Oliver et al. 2014; Ballinger and Perlman 2019). In insects, vertical transmission of bacterial symbionts places heritable symbionts into direct conflict with the natural enemies of their host. This conflict has driven the evolution of host protection in a number of symbiont clades, in a wide range of host species, against a diverse range of enemies. For example, microbial symbionts are known to provide protection against ssRNA viruses (Hedges et al. 2008; Teixeira et al. 2008), nematodes (Jaenike et al. 2010), fungal pathogens (Scarborough et al. 2005; Lukasik et al. 2013) and parasitic wasps (Oliver et al. 2003; Xie et al. 2010, 2014; Mateos et al. 2016; Paredes et al. 2016; Ballinger and Perlman 2017).

Studies of defensive symbiosis are most well developed in aphid–symbiont–enemy interactions. For example, in the black bean aphid (*Aphis fabae*), the level of resistance conferred against the parasitoid (*Lysiphlebus fabarum*) is dependent on the interaction between the strain of defensive symbiont (*Hamiltonella defensa*) and the strain of the parasitoid, not the host itself (Schmid et al. 2012; Cayetano and Vorburger 2013, 2015). Similarly, in the pea aphid (*Acyrthosiphon pisum*), protection against the entomopathogenic fungus *Pandora neoaphidis*, is strongly dependent on the genotype–by–genotype interaction between the parasite and the defensive facultative symbiont, *Regiella insecticola* (Parker et al. 2017). Although these studies have demonstrated the importance of heritable microbes in mediating host-parasite specificity, the generality of these interaction terms is yet to be determined beyond the aphid system.

Regarded as a historically important model system for defence ecology and evolution, symbiont-mediated protection also occurs in the genus *Drosophila*. The facultative endosymbiont, *Spiroplasma*, can protect *Drosophila* against a range of endoparasitoid wasps. In *Drosophila hydei*, the native *Spiroplasma* strain Hy1 protects flies from the endoparasitoid wasp, *Leptopilina heterotoma* (Xie et al. 2010), although wasp attack survivors are found to have reduced fertility (Xie et al. 2011). Similarly, in *Drosophila melanogaster*, the *Spiroplasma* strain MSRO protects flies attacked by *Leptopilina boulardi* (Xie et al. 2014; Paredes et al. 2016; Ballinger and Perlman 2017), *Leptopilina victoriae* and *Ganapis xanthopoda* (Mateos et al. 2016). In *Drosophila neotestacea*, *Spiroplasma* confers tolerance against *Howardula* nematode worms, rescuing the fertility of female fly hosts (Jaenike et al. 2010).

Despite their importance as a model system, our understanding of *Spiroplasma*-mediated protection in *Drosophila* is limited in comparison to the equivalent aphid systems. Exploration of evolutionary dynamics is limited to the observation of the sweep of protective symbionts through North American *D. neotestacea* over time (Jaenike et al. 2010). More attention has been given to establishing the extent and molecular underpinnings of the defensive mechanisms. Variation in protective capacity against different parasitoid natural enemies has been observed. For example, whilst *Spiroplasma* strain MSRO is only very weakly able to rescue *D. melanogaster* flies parasitised by *L. heterotoma*, the same symbiont strain increases fly survival by 50% against *L. boulardi* (Xie et al. 2014; Paredes et al. 2016; Ballinger and Perlman 2017). Defence is considered mechanistically to occur through a combination of RIP toxins secreted by the symbiont, and competition between symbiont and wasp for lipid (Paredes et al. 2016, Ballinger and Perlman 2017).

To date, *Spiroplasma* defence of *Drosophila* against natural enemy attack has commonly been examined in a coarse-grained fashion, with protection against one strain of any particular enemy species being assessed. Parallels with the aphid system indicate there may be more subtle interactions with enemy genotype, such that measures of protection against one enemy strain do not necessarily reflect the outcome of all interactions with members of that species. Further, symbiont-mediated defences have been commonly treated in isolation of other defence systems. Previous work has shown that environmental ethanol is an important determinant of the outcome of parasitoid wasp attack in *Drosophila*, with consumption of ethanol by infected larvae increasing mortality of wasp larvae growing within the haemocoel (Milan et al. 2012; Lynch et al. 2017). This observation implies that the magnitude of protection against wasp attack afforded by symbionts should be measured across a range of environmental ethanol conditions, to improve our ability to predict the outcome of the interaction, and from this, symbiont dynamics.

Understanding the dynamics of symbiont-mediated defence in natural populations thus requires us to determine variation in *Spiroplasma*-mediated protection across enemy strains, and assess how this interacts with other protective mechanisms such as ethanol-mediated protection. In this study, we therefore assessed whether the variation in *Spiroplasma*-mediated protection previously observed against different wasp species is reflected also in variation in protection against different strains of the same wasp species. Furthermore, we examined whether the degree of protection and specificity to parasite strain, are altered by ethanol presence. Within this study, we combine fly survival data with data on the fertility of flies that survived wasp attack to establish a protective index (PI) for each combination, which represents the first composite measure of symbiont-mediated protection obtained in any system to date.

## Materials and methods

### Insect strains and maintenance

*D. melanogaster* Canton-S flies with and without *Spiroplasma* MSRO-infected Red 42 were used. MSRO-infected Red 42 were originally collected in Brazil in 1997 and maintained in the lab in a Canton-S background in parallel to Canton-S control stock lacking *Spiroplasma*, from which males were derived each generation for MSRO line maintenance (Montenegro et al. 2000). This strain has previously been shown to kill Lh14 wasps, but produces very weak fly survival (Xie et al. 2014; Ballinger and Perlman 2017). These stocks both carried *Wolbachia* strain wmelCS, which occurs naturally and does not affect protection (Xie et al. 2014). It should be noted that all larvae from the *Spiroplasma*-infected treatments are female due to the high efficiency of male-killing. However, there does not appear to be any differences in survival between the sexes against parasitoid wasp attack (Xie et al. 2014). All flies were maintained on Corn Meal Agar (10 g agarose, 85 g sugar, 60 g maize meal, 40 g autolysed yeast in a total volume of 1 L, to which 25 mL 10% Nipagin was added) at 25 °C on a 12:12 light:dark cycle.

The *L. heterotoma* used were an inbred strain collected from Sainte Foy-lès-Lyon and la Voulte, France, a strain caught in Madeira, Portugal in March 2017, and the inbred strain Lh14 used in previous studies, initially collected in Winters, California in 2002 (Schlenke et al. 2007). All wasp strains tested positive for *Wolbachia*. Wasp stocks were maintained on second instar Oregon-R larvae at 25 °C on a 12:12 light:dark cycle. After emergence wasps were maintained in grape agar vials, supplemented with a Flug® (Genesee Scientific) moistened with honey water and allowed to mature and mate for 7 days prior to exposure to *D. melanogaster* L2 larvae.

### Preparing ethanol food

The wasp attack assay was performed in fly medium at 0 and 6% ethanol, which is within the normal range experienced by *D. melanogaster* larvae in nature (McKenzie and McKechnie 1979; Gibson et al. 1981). Medium was prepared by using the standard Corn Meal Agar recipe (above) with the exception of the quantity and concentration of Nipagin added (5 mL 50% w/v/1 L of medium), to ensure the concentration of ethanol in the experimental vials was close to 0 and 6%. To prevent the evaporation of ethanol during the process, 200 mL of food was dispensed into 250 mL Duran bottles and allowed to cool to 45 °C before 12 mL of 100% ethanol was added to the ethanol treatment bottles and homogenised. Six millilitres of food was then dispensed into standard *Drosophila* vials and instantly covered with Parafilm to prevent ethanol evaporation before experimental larvae were transferred into the vials.

### Wasp attack assay

To ensure efficient vertical transmission of *Spiroplasma*, MSRO-infected Red 42 females were aged to at least 10 days prior to egg laying. Flies were allowed to mate in cages and lay eggs on a grape Petri dish painted with live yeast for 24 h. Grape Petri dishes were incubated for a further 24 h to allow larvae to hatch. First instar larvae were picked from the grape plate into the experimental vials at 30 larvae per vial. Eight treatments were formed per wasp strain with ~10–15 replicate vials per treatment (1) Lh− S− EtOH−, (2) Lh− S− EtOH+, (3) Lh− S+ EtOH−, (4) Lh− S+ EtOH+, (5) Lh+ S− EtOH−, (6) Lh+ S−EtOH+, (7) Lh+ S+ EtOH−, (8) Lh+ S+ EtOH+. Five experienced female wasps and three male wasps were transferred into the wasp treatment vials. Flugs® (Genesee Scientific) were used to bung vials to reduce ethanol evaporation. Adult wasps were allowed to parasitise for 2 days before being removed. All vials were maintained at 25 °C on a 12:12 light:dark cycle. For each vial, the number of pupae, emerging flies, and emerging wasps were recorded.

### Measuring fertility

To determine the degree to which the survivors of wasp attack were impacted by wasp attack, the average daily emerged offspring of *Spiroplasma*-infected survivors (‘exposed’) and *Spiroplasma*-infected flies, which did not undergo wasp attack (‘unexposed’) were measured from both the 0 and 6% ethanol treatments. Only fly survivors that underwent attack from the *L. heterotoma* Lh-Fr and Lh-Mad strains were used, as there were few survivors from the attack of the Lh14 strain of *L. heterotoma* and very low numbers also from the *Spiroplasma*-uninfected wasp attacked group.

To this end, adult female flies from the wasp attack assay were retained on eclosion, and stored in vials containing sugar yeast medium (20 g agarose, 100 g sugar, 100 g autolysed yeast in a total volume of 1 L, to which 30 mL 10% Nipagin w/v propionic acid was added) at mixed ages. A week after emergence commenced, ~45 female flies from each of the *Spiroplasma* treatments were placed individually into a vial containing 6 mL of Corn Meal Agar with two Canton-S males with a single yeast ball and allowed to mate. These flies were transferred onto fresh vials each day for 5 days. Female fertility was measured as the average number of daughters produced over 4 days (day 2–5), with F1 flies given 2 weeks to emerge to ensure every fly had emerged before counting. Females which did not produce any daughters were considered infertile.

### Measuring wing size

Body size as adult measures the stress experienced by flies during development, with many stressors (density, ethanol) resulting in smaller adult flies (Miller and Thomas 2006; Castañeda and Nespolo 2013). To determine whether wasp attack affected female body size, wing size was used as a proxy, as these factors are known to be highly correlated in *Drosophila* (Robertson and Reeve 1952). To this end, the left wings of individual flies from the experiment above were removed using forceps under a microscope (right wings were used if left wings were damaged) and mounted flat onto a glass microscope slide. A photograph was taken of each wing using a microscope mounted camera using GXCapture-O software (6.9 v). Using the ImageJ software (1.49 v, US National Institutes of Health, USA), the area of the wing was determined by locating the coordinates of the six wing landmarks as defined in Gilchrist and Partridge (2001) and calculating the interior area of the polygon created. A scale slide was used to transform all wing measurements into millimetre square units. All photos where the landmarks were not clearly visible were not measured and excluded from the analysis.

### Wasp strain oviposition

To determine whether the differences in fly survival were due to differences in wasp oviposition behaviour, we compared the number of wasp eggs and larvae per fly larva among the three wasp strains (Lh-Fr, Lh14 and Lh-Mad). In addition, we determined whether wasp oviposition differed between *Spiroplasma* positive and negative fly larvae. To this end, we followed the same protocol as the wasp attack assay, except the no-wasp control and 6% ethanol treatment was omitted. Immediately after wasp removal, ~5 fly larvae from each of the five replicate vials were dissected under a microscope to count the number of wasp eggs and/or larvae present.

### Statistical analysis

All statistical analyses were performed using the statistical software R, version 3.5.0 (R Development Core Team 2018). Fly and wasp survival, proportion of flies fertile, and wasp oviposition were analysed by fitting a generalized linear model with binomial, binomial and Poisson distributions, respectively. A Bayesian generalized linear model (‘bayesglm’ function in the ‘arm’ package; Gelman et al. 2018) was used to analyse wasp survival due to extreme separation between symbiont treatments (*Spiroplasma* positive treatments had zero wasp survival), and for this reason, symbiont interaction terms were additionally excluded from the analysis. The number of daughters produced and fly wing size were analysed using linear models. Wing area measurements were Box-Cox transformed to conform to normality (Crawley 2007). In all cases, a fully saturated model including all factors and their interaction was reduced to a minimum adequate model through step-wise simplification. Nonsignificant factors are reported as the output of the model comparisons. The effect of significant independent variables are reported from the analysis of the minimum adequate model using the ‘car’ package.

To produce a composite measure of protection, a PI was calculated by comparing the survival and fecundity of *Spiroplasma*-infected flies in the presence/absence of a given strain of wasp. The PI was calculated as the ratio of p(survival) × p(fertile) × fecundity of fertile individuals for attacked vs unattacked *Spiroplasma*-infected flies and reflects the benefit of *Spiroplasma* in the face of wasp attack. Credible intervals for PI were calculated through simulation. By assuming prior probability distributions for each parameter (survival probability = beta distribution; fertility probability = beta distribution; fecundity = normal distribution), the ‘rbeta’ and ‘rnorm’ functions were used to calculate 95% credible intervals for PI. The simulation data was also used to establish the posterior probability of PI differing between attacking wasp strains.

## Results

### Fly survival and wasp success

**Fig. 1:**
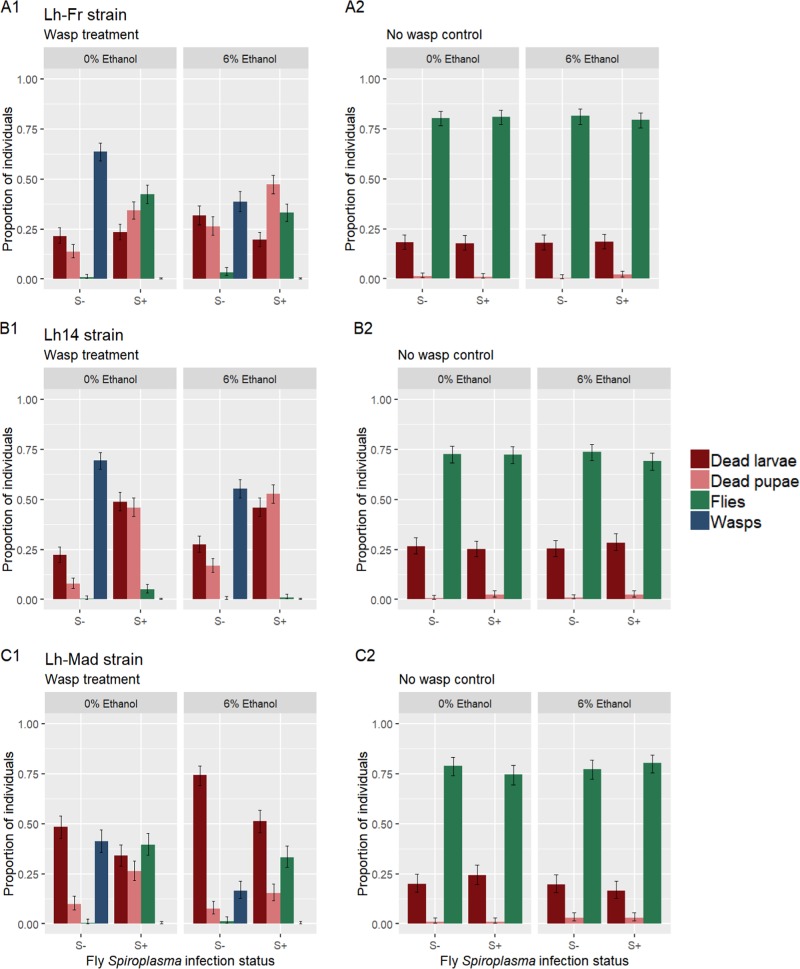
Proportion of dead larvae (red), dead pupae (pink), emerging flies (green) and emerging wasps (blue) for *Spiroplasma*-infected and -uninfected *Drosophila melanogaster* attacked by three different *Leptopilina heterotoma* strains in 0 and 6% environmental ethanol. Error bars represent 95% binomial confidence intervals.

In the absence of *L. heterotoma*, mean larva-to-adult fly survival was >69% across all treatments (Fig. 1). There was no significant effect of *Spiroplasma* (*χ*^2^ = 0.990, d.f. = 1, *P* = 0.320) or ethanol (*χ*^2^ = 0.00820, d.f. = 1, *P* = 0.928), nor a significant interaction between *Spiroplasma* and ethanol on fly larva-to-adult survival (*χ*^2^ = 0.0625, d.f. = 1, *P* = 0.803).

In the presence of *L. heterotoma*, fly *Spiroplasma* infection had a significantly strong and positive effect on fly larva-to-adult survival (*χ*^2^ = 223, d.f. = 1, *P* < 0.001; Fig. 1). The effect of *Spiroplasma* on fly larva-to-adult survival depended on the strain of attacking parasitoid, which was reflected in a significant interaction between *Spiroplasma* and wasp strain (*χ*^2^ = 9.64, d.f. = 2, *P* = 0.008). *Spiroplasma* provided almost no protection against the Lh14 strain of *L. heterotoma*, increasing fly larva-to-adult survival slightly from <1 to 5.11%. *Spiroplasma* did however, provide strong protection against the Lh-Fr and Lh-Mad wasp strains, increasing fly larva-to-adult survival from <1% to 42.4% and 39.7% respectively. Wasp strain itself had a significant effect on fly larva-to-adult survival (*χ*^2^ = 191.02, d.f. = 2, *P* < 0.001).

The presence of ethanol had a weak, albeit significant positive effect on fly larva-to-adult survival in the presence of wasps (*χ*^2^ = 10.3, d.f. = 1, *P* = 0.001; Fig. 1). However, the effect of ethanol differed between the strains of attacking *L. heterotoma*, which was reflected in a significant interaction between ethanol and wasp strain (*χ*^2^ = 7.82, d.f. = 2, *P* = 0.020). Specifically, the presence of ethanol in the absence of *Spiroplasma* reduces fly larva-to-adult survival against the Lh14 *L. heterotoma* strain from 0.45 to 0.22%, yet slightly increases fly larva-to-adult survival against the Lh-Fr strain from 0.89 to 3.33% and the Lh-Mad strain from 0.33 to 1.33%. There was also a significant interaction between *Spiroplasma* and ethanol (*χ*^2^ = 11.3, d.f. = 1, *P* *<* 0.001; Fig. 1), with the presence of ethanol reducing the effect of *Spiroplasma*-mediated fly larva-to-adult survival across all three wasp strains (% decrease; Lh-Fr = 22%, Lh14 = 78%, Lh-Mad = 16%). The interaction between *Spiroplasma*, wasp strain and ethanol was not found to be significant (*χ*^2^ = 0.365, d.f. = 2, *P* = 0.833).

Wasp success was strongly negatively affected by fly *Spiroplasma* infection, with the presence of *Spiroplasma* completely preventing the emergence of wasps across all *L. heterotoma* strains in both the presence and absence of ethanol (*χ*^2^ = 23.5, d.f. = 1, *P* < 0.001). In the absence of *Spiroplasma*, the presence of ethanol had a significantly negative effect on wasp success (*χ*^2^ = 102, d.f. = 1, *P* < 0.001; Fig. 1). However, the effect of ethanol depended on the strain of attacking *L. heterotoma*, reflected in a significant interaction between ethanol and wasp strain (*χ*^2^ = 8.42, d.f. = 2, *P* *=* 0.015). Ethanol reduced wasp success by 40%, 21%, and 60% across the Lh-Fr, Lh14 and Lh-Mad strains, respectively. Wasp success was also significantly affected by the strain of wasp (*χ*^2^ = 154, d.f. = 2, *P* < 0.001).

### Female fertility

#### Proportion fertile

**Fig. 2:**
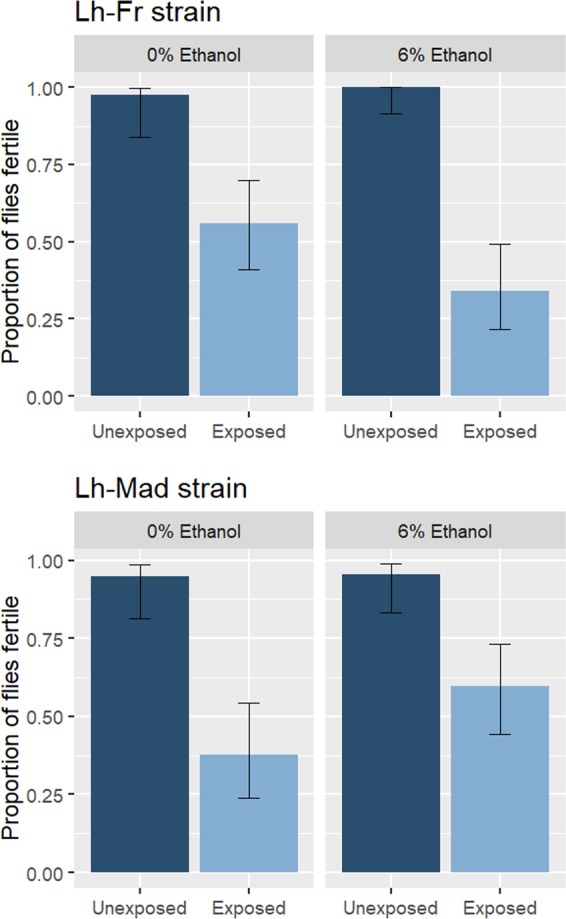
The proportion of *Spiroplasma*-infected *Drosophila melanogaster* females considered fertile after exposure to *Leptopilina heterotoma* (Lh-Fr and Lh-Mad strain) and unexposed controls developed through 0 and 6% ethanol medium. Dark blue bars indicate unexposed controls and light blue bars represent wasp exposed. Error bars represent 95% binomial confidence intervals.

For both Lh-Fr and Lh-Mad attacking wasp strains, *Spiroplasma*-infected individuals that survived wasp attack were observed to have reduced fertility, measured as the proportion of females able to produce progeny (Fig. 2).

For attack with the Lh-Fr strain of wasp, there was a significant effect of wasp attack on the proportion of flies which were found to be fertile (*χ*^2^ = 19.8, d.f. = 1, *P* < 0.001; Fig. 2). The proportion of *D. melanogaster* considered fertile following the wasp attack was reduced by 55% compared with control non-attacked *D. melanogaster*. There was no significant effect of ethanol (*χ*^2^ = 3.11, d.f. = 1, *P* = 0.078), nor a significant interaction between ethanol and wasp attack (*χ*^2^ = <0.001, d.f. = 1, *P* = 0.988).

For attack with the Lh-Mad strain, there was a significant effect of wasp attack on the proportion of flies found to be fertile (*χ*^2^ = 28.4, d.f. = 1, *P* < 0.001; Fig. 2). The proportion of *D. melanogaster* considered fertile following the wasp attack was reduced by 48% compared with control non-attacked *D. melanogaster*. There was no significant effect of ethanol (*χ*^2^ = 3.23, d.f. = 1, *P* = 0.072), nor a significant interaction between ethanol and wasp attack (*χ*^2^ = 0.447, d.f. = 1, *P* = 0.504).

#### Number of daughters produced

**Fig. 3:**
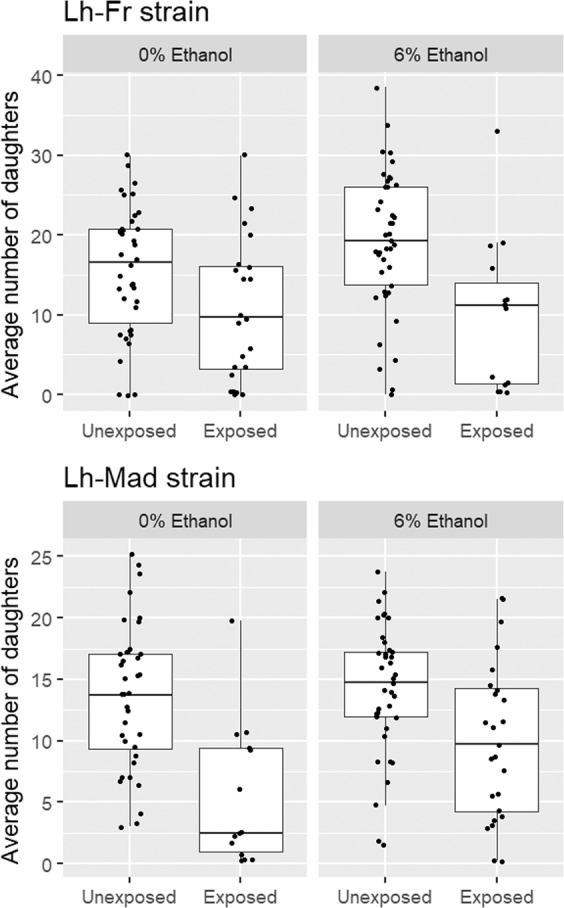
The average number of daughters produced by fertile *Spiroplasma*-infected female *Drosophila melanogaster* exposed to *Leptopilina heterotoma* (Lh-Fr and Lh-Mad strain) and unexposed controls developed through 0 and 6% ethanol medium. The box plots display the upper and lower quartiles, the median and the range. Points represent each measurement obtained.

In both cases, *Spiroplasma*-infected individuals that survived wasp attack and were fertile were observed to produce fewer daughters compared with fertile, unattacked controls (Fig. 3).

For attack with the Lh-Fr strain, wasp attack significantly reduced the average number of daughters produced with protected wasp attacked *D. melanogaster* averaging ~39% fewer than control unattacked *D. melanogaster* (Mean ± SE = 10.6 ± 1.44 daughters for attacked flies vs. 17.5 ± 0.969 daughters for control flies; *F* = 16.4, d.f. = 1116, *P* < 0.001; Fig. 3). There was no significant effect of ethanol (*F* = 1.81, d.f. = 1115, *P* *=* 0.181), nor a significant interaction between ethanol and wasp attack (*F* = 1.74, d.f. = 1114, *P* *=* 0.190).

For attack with the Lh-Mad strain, wasp attack also significantly reduced the average number of daughters produced with wasp attacked protected *D. melanogaster* averaging ~40% fewer than control *D. melanogaster* (Mean ± SE = 8.35 ± 1.04 daughters for attacked flies vs. 14.0 ± 0.626 daughters for control flies; *F* = 23.9, d.f. = 1114, *P* < 0.001; Fig. 3). There was no significant effect of ethanol (*F* = 2.9, d.f. = 1113, *P* *=* 0.094), nor a significant interaction between ethanol and wasp attack (*F* = 2.9, d.f. = 1112, *P* *=* 0.092).

### Overall protection

Taking into account the survival, proportion of adults fertile, and the fecundity of wasp attack survivors, compared with unexposed *Spiroplasma*-infected controls, a PI was calculated as the product of fly survival × p(fertile) × fecundity of exposed vs unexposed *Spiroplasma*-infected flies (this metric assumes complete mortality from wasps in the absence of *Spiroplasma*, which is approximately true as <1% of individuals tested survived wasp attack). In the absence of ethanol, the estimated protection index was 21%, and 9% against the Lh-Fr and Lh-Mad strains respectively (Table 1). The posterior probability that the protection index for *Spiroplasma* against the Lh-Fr strain is greater than the protection index against the Lh-Mad strain was 0.99. In contrast, the PI in the presence of ethanol was 7% and 12% against Lh-Fr and Lh-Mad wasp strains respectively (Table 1). The posterior probability that the protection index for *Spiroplasma* against the Lh-Mad strain is greater than the protection index against the Lh-Fr strain in the presence of ethanol was 0.99. With no fecundity measure available for Lh14 (due to insufficient survivors), we assume the estimate of protection to be less than the survival value.

**Table 1:** The overall protection conferred by *Spiroplasma* against the Lh-Fr, Lh14 and Lh-Mad *Leptopilina heterotoma* strains in *Drosophila melanogaster* in the absence (A) and presence of ethanol (B).

**A**) In the absence of ethanol					
Wasp strain	Treatment	Fly survival (binomial 95% CI intervals (lower, upper))	Proportion fertile (binomial 95% CI intervals (lower, upper))	Fecundity measure ± SE	Estimated protective index (95% credible interval (lower, upper))
Lh-Fr	Exposed S−	<0.01 (0.0033–0.023)	N/A	N/A	0.21 (0.12, 0.33)
	Exposed S+	0.42 (0.38–0.47)	0.56 (0.41– 0.70)	10.9 ± 1.83	
	Unexposed control S+	0.81 (0.77–0.84)	0.97 (0.84– 0.99)	15.6 ± 1.31	
Lh14	Exposed S−	<0.01	N/A	N/A	<0.036
	Exposed S+	0.05	N/A	N/A	
	Unexposed control S+	0.72	N/A	N/A	
Lh-Mad	Exposed S−	<0.01 (0.00047–0.023)	N/A	N/A	0.09 (0.033, 0.16)
	Exposed S+	0.40 (0.34–0.45)	0.40 (0.24–0.54)	5.45 ± 1.54	
	Unexposed control S+	0.75 (0.69–0.79)	0.95 (0.81–0.99)	13.7 ± 0.985	
**B**) In the presence of 6% ethanol					
Wasp strain	Treatment	Fly survival (binomial 95% CI intervals (lower, upper))	Proportion fertile (binomial 95% CI intervals (lower, upper))	Fecundity measure ± SE	Estimated protective index (95% credible intervals (lower, upper))
Lh-Fr	Exposed S−	0.03 (0.019, 0.058)	N/A	N/A	0.07 (0.033, 0.13)
	Exposed S+	0.33 (0.29, 0.38)	0.34 (0.22, 0.49)	9.98 ± 2.41	
	Unexposed control S+	0.80 (0.76, 0.83)	1 (0.91, 1.00)	19.2 ± 1.38	
Lh14	Exposed S−	<0.01	N/A	N/A	<0.007
	Exposed S+	0.01	N/A	N/A	
	Unexposed control S+	0.69	N/A	N/A	
Lh-Mad	Exposed S−	0.01 (0.0050, 0.035)	N/A	N/A	0.12 (0.12, 0.27)
	Exposed S+	0.33 (0.28, 0.39)	0.60 (0.44, 0.73)	6.38 ± 1.28	
	Unexposed control S+	0.80 (0.75, 0.84)	0.95 (0. 83, 0.99)	14.2 ± 0.805	

Exposed S− = wasp attacked *Spiroplasma*-uninfected flies; Exposed S+ = wasp attacked *Spiroplasma*-infected flies; Unexposed S+ = *Spiroplasma*-infected flies not attacked. Protective Index is calculated as [p(survival) × p(fertile) × fecundity of fertile individuals] of exposed S+ vs unexposed control S+ individuals with credible intervals calculated as given in methods

### Wing size

**Fig. 4:**
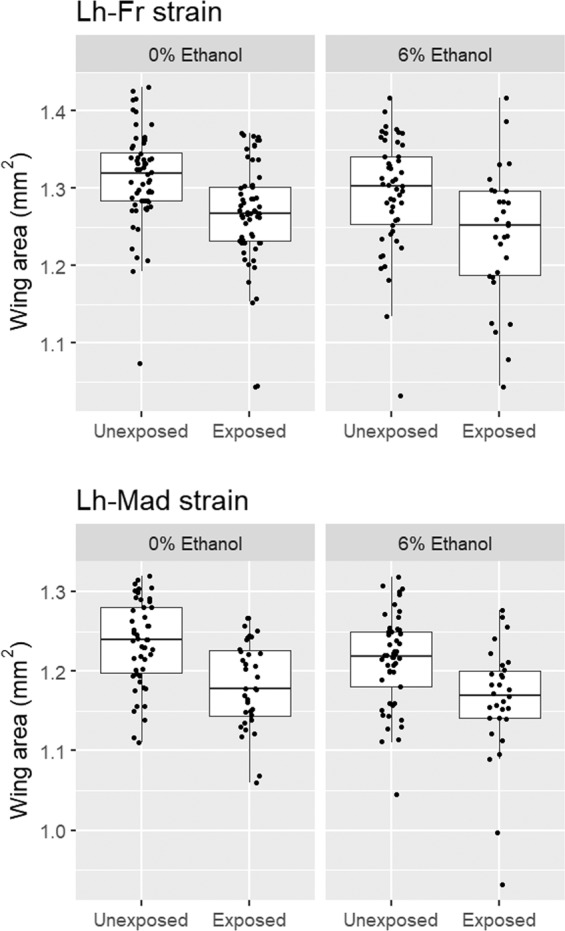
The wing area (mm^2^) of *Spiroplasma*-infected female *Drosophila melanogaster* exposed to *Leptopilina heterotoma* (Lh-Fr and Lh-Mad strain) and unexposed controls developed through 0 and 6% ethanol medium. The box plots display the upper and lower quartiles, the median and the range. Points represent each measurement obtained.

In both cases, *Spiroplasma*-infected individuals that survived wasp attack, were smaller compared with unattacked *Spiroplasma*-infected individuals (Fig. 4).

For attack with the Lh-Fr wasp strain, wasp attack strongly reduced wing size, with the wings of wasp attacked female *D. melanogaster* on average 0.04 mm^2^ (3%) smaller than unattacked *D. melanogaster* (Mean ± SE = 1.26 ± 0.008 mm^2^ for attacked flies vs. 1.30 ± 0.006 mm^2^ for unattacked flies; *F* = 26.7, d.f. = 1196, *P* < 0.001; Fig. 4). Ethanol reduced wing size, with the wing size of *D. melanogaster* reared in ethanol on average 0.02 mm^2^ (1.6%) smaller than *D. melanogaster* reared in the absence of ethanol (Mean ± SE = 1.27 ± 0.008 mm^2^ for flies reared in 6% ethanol vs. 1.29 ± 0.007 mm^2^ for control flies; *F* = 4.34, d.f. = 1196, *P* = 0.038; Fig. 4). There was no significant interaction between ethanol and wasp attack on wing size (*F* < 0.001, d.f. = 1195, *P* = 0.980).

For attack with the Lh-Mad wasp strain, wasp attack also had a highly significant effect on wing size, with the wing size of wasp attacked female *D. melanogaster* on average 0.05 mm^2^ (4%) smaller than control *D. melanogaster* (Mean ± SE = 1.17 ± 0.006 mm^2^ for attacked flies vs. 1.22 ± 0.006 mm^2^ for unattacked flies; *F* = 31.9, d.f. = 1162, *P* < 0.001; Fig. 4). Ethanol reduced wing size, with the wing size of *D. melanogaster* reared in ethanol on average 0.02 mm^2^ (1.7%) smaller than *D. melanogaster* reared in the absence of ethanol (Mean ± SE = 1.19 ± 0.068 mm^2^ for flies reared in 6% ethanol vs. 1.21 ± 0.007 mm^2^ for control flies; *F* = 4.71, d.f. = 1,162, *P* = 0.032; Fig. 4). There was no significant interaction between ethanol and wasp attack on wing size (*F* = 0.227, d.f. = 1161, *P* = 0.634).

### Wasp oviposition

**Fig. 5:**
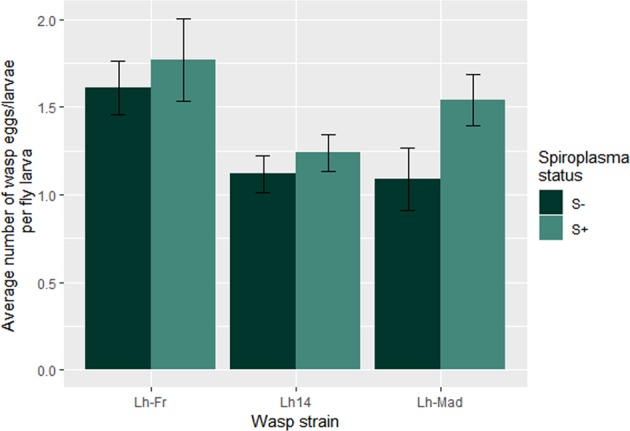
The average number of wasp eggs/larvae in *Spiroplasma* positive and negative *Drosophila melanogaster* larvae following 48 h of parasitisation by three strains of *Leptopilina heterotoma*. Dark green bars indicate *Spiroplasma* negative individuals and light green bars represent *Spiroplasma* positive individuals. Error bars depict ± SE.

The average number of wasp eggs laid into a fly larva across a 48 h period of parasitisation was >1 but <2 for all treatments (Fig. 5). There was no significant effect of wasp strain (*χ*^2^ = 4.94, d.f. = 2, *P* = 0.085) or fly *Spiroplasma* infection status (*χ*^2^ = 1.52, d.f. = 1, *P* = 0.218), nor a significant interaction between wasp strain and fly *Spiroplasma* infection (χ^2^ = 0.664, d.f. = 2, *P* *=* 0.718) on the number of wasp eggs laid into fly larvae.

## Discussion

It is now recognised that the outcome of natural enemy attack can be determined by the presence or absence of defensive heritable symbionts. Beyond their presence, the outcome of these interactions can also depend on the genotypes of all players: symbiont, host and enemy. However, the specificity of symbiont-mediated defence has only been explored within the aphid system. Previous work has found wasp species to be an important component of *Spiroplasma*-mediated protection in *Drosophila*, with *Spiroplasma* able to protect against some wasp species, but not others (Mateos et al. 2016). Protection against *L. heterotoma*, using strain Lh14, for instance, is considered weak or absent in three previous studies (Xie et al. 2014; Paredes et al. 2016; Ballinger and Perlman 2017). In this study, we examined whether protection against *L. heterotoma* wasps varied with wasp strain. Protection against the Lh14 wasp strains was observed at the low level previously recorded. In contrast, substantial protection was exhibited against the other strains of *L. heterotoma*. The overall protection gained by harbouring *Spiroplasma* against the Lh-Fr, Lh-Mad and Lh14 *L. heterotoma* was ~21%, 9% and <4% respectively, measured in the absence of environmental ethanol. Thus, *Spiroplasma* is protective against *L. heterotoma*, but the degree of protection is wasp strain dependent.

The differences in protective index afforded by *Spiroplasma* against different wasps strains arose through both effects on survival in response to wasp attack (Lh14 attack kills flies notwithstanding *Spiroplasma* presence, whereas *Spiroplasma* rescues flies attacked by Lh-Mad/Lh-Fr strains) and through differences in fertility/fecundity (between flies surviving attack by Lh-Fr and Lh-Mad strains). Thus, we conclude that the protection afforded by *Spiroplasma* against *L. heterotoma* is dependent on *L. heterotoma* genotype, and that the differences observed are a product of both fly survival and survivor fertility differences. We would note that whilst impacts on the fertility/fecundity of ‘protected’ survivors of attack is noted in some cases of defensive symbiosis (Xie et al. 2011; Vorburger et al. 2013), these metrics have not previously been included in models of relative protection against different enemy strains/species. Our data indicate that a complete model of protection dynamics may require measurement and inclusion of these parameters.

The mechanistic processes that determine the degree to which *Spiroplasma* affords protection against different wasp genotypes are uncertain. *Spiroplasma* completely prevented any wasps from emerging in all cases, implying that the symbiont defensive system was always efficient at killing the wasp. However, the degree to which killing the wasp rescued their fly host varied. Flies could be seen developing in the pupal cases in the majority of cases, but it was variation in eclosion to adult that underlies differential fly survival in response to the different genotypes of wasp. Further, we observed variation in surviving fly fertility that implies varying damage from the wasp is carried over beyond the point of wasp death, potentially associated with the physical consumption of the fly during parasitisation. From the wasp differential oviposition assay, we can reject the hypothesis that the observed differences are due to differential oviposition behaviour across wasp strains.

The origins of differential fly survival therefore lie within a parasitised host individual. What is it about the wasp–host interaction in the presence of *Spiroplasma* that leads to different outcomes in terms of fly survival? One possibility is that RIP toxins involved in protection differentially affect the wasp strains studied, with Lh14 being less sensitive to RIPs, and thus developing further and/or causing more damage to the fly. A second explanation is the variation in the ability of the wasp to synthesise lipids, for which the *Spiroplasma* is thought to compete (Paredes et al. 2016). Intraspecific variation in the ability to synthesise lipids has been observed in *L. heterotoma* (Visser et al. 2018). If Lh14 is unable to synthesise lipids, this could lead to competition between *Spiroplasma* and the wasp for the available lipids within the host, thus leading to lower survival. A third, non-mutually exclusive, explanation is that the different outcomes are a result of variation in the venom transferred by the wasp strains. Venom is transferred along with eggs to suppress the host immune system and bypass nuclear encoded defences. In this model, a wasp strain delivering more potent venom can develop further or, causes damage that prevents fly survival. Intraspecific venom variation amongst *Leptopilina* wasps is known (Colinet et al. 2013). Wasp venom evolution has also been suggested as the target of selection when a wasp is passaged through symbiont-protected aphids (Dennis et al. 2017). This study indicates that the venom constitution is likely to be important in determining the outcome of a wasp–host interaction in the presence of symbionts. Two open questions therefore remain. First, what is the aspect of the wasp (sensitivity to RIP toxins, lipid synthesis, venom, other) that is important in producing the variation in protection afforded by *Spiroplasma*? Second, are changes in fly survival associated with longer development of the wasp, or more damage created by certain wasps (with similar total development)? These await further research.

The protection offered by *Spiroplasma* against wasp strains is modified by the presence of environmental ethanol during the larval phase. In contrast to assays where ethanol was absent, protection in the presence of ethanol is strongest against the Lh-Mad strain of wasp, and less strong against the Lh-Fr strain, with protection absent against Lh14. Against the Lh-Fr wasp strain, ethanol had a negative effect on the overall *Spiroplasma*-mediated fly protection, reducing protection from 21 to 7%. In contrast, ethanol had a positive effect on the overall protection against the Lh-Mad wasp strain, increasing overall protection from 9 to 12%, mainly due to the presence of ethanol reducing the negative effect of wasp attack on survivor fertility. In all cases, ethanol was detrimental to fly survival upon wasp attack. These results indicate that the interaction between *Spiroplasma*-mediated protection and ethanol protection is dependent on the genotype of the attacking wasp.

The data presented here have significant implications for the evolutionary and ecological dynamics of the *Spiroplasma*-*Drosophila*-wasp tripartite interaction in natural populations. From the perspective of the symbiont, the fitness benefit of protection is dependent upon wasp genotype, and thus the degree to which wasp attack drives the symbiont to higher prevalence will depend on the profile of the wasp population. In contrast, the observation that wasp emergence is zero in the presence of the symbiont in all three cases, implies that the symbiont will not select upon the wasp population directly, although it will decrease the size of this population. However, a caveat here is that our results are derived from three wasp strains and their interaction with one *Spiroplasma* isolate. It is possible other wasp strains are resistant to *Spiroplasma*, and that there are strains of *Spiroplasma* which are less efficient at killing wasps.

Environmental ethanol, which modulates wasp attack outcome, is likely to be less important than *Spiroplasma*-mediated protection in terms of determining wasp success. In contrast to other lab studies (Milan et al. 2012; Kacsoh et al. 2013; Lynch et al. 2017), here we observed only a small magnitude of protection afforded by ethanol alone. Possible reasons for the disparity include variation in fly strains (Canton-S here, Oregon-R in other studies) and differences in experimental protocols (e.g. the period of exposure). Nevertheless, ethanol did determine the relative protective benefit of *Spiroplasma* against different wasp strains. Thus, the presence/absence of ethanol melds with the genetic makeup of the wasp population to determine protection accorded by *Spiroplasma*, and ultimately therefore is predicted to impact *Spiroplasma* dynamics.

In summary, our work has extended the aphid synthesis to *Drosophila*, and indicates symbiont-mediated protection appears generally to depend on the genotype of the attacking wasp species. Further the environment (in this case ethanol) may modulate protection. More widely, it will be important not to disregard other protective mechanisms and their interaction when predicting the ecological dynamics of symbiont-mediated protection in this model system. Indeed, how *Spiroplasma*-mediated protection is predicted to interact with *Drosophila’s* own innate immunity (and more widely, host genetic background) requires further investigation. Beyond this, parallels with studies of aphids indicate that the symbiont genotype and environment should be considered. Thermal environment, for instance, commonly affects the symbiotic phenotype, and low temperatures are known to ablate *Spiroplasma* male-killing (Anbutsu et al. 2008). Thus, whilst our study indicates the presence of complex interaction terms in this tripartite interaction, the full extent of these awaits resolution.

## Supplementary information

Table S1

## Data Availability

Data generated and analysed during this study are available at figshare (10.6084/m9.figshare.c.4551422.v1).
